# Seasonal Influenza Vaccine and Protection against Pandemic (H1N1) 2009-Associated Illness among US Military Personnel

**DOI:** 10.1371/journal.pone.0010722

**Published:** 2010-05-19

**Authors:** Matthew C. Johns, Angelia A. Eick, David L. Blazes, Seung-eun Lee, Christopher L. Perdue, Robert Lipnick, Kelly G. Vest, Kevin L. Russell, Robert F. DeFraites, Jose L. Sanchez

**Affiliations:** 1 Division of GEIS Operations, Armed Forces Health Surveillance Center, Silver Spring, Maryland, United States of America; 2 Henry M. Jackson Foundation for the Advancement of Military Medicine, Armed Forces Health Surveillance Center, Silver Spring, Maryland, United States of America; 3 Division of Communication, Standards and Training, Armed Forces Health Surveillance Center, Silver Spring, Maryland, United States of America; 4 Armed Forces Health Surveillance Center, Silver Spring, Maryland, United States of America; Erasmus Medical Center, Netherlands

## Abstract

**Introduction:**

A novel A/H1N1 virus is the cause of the present influenza pandemic; vaccination is a key countermeasure, however, few data assessing prior seasonal vaccine effectiveness (VE) against the pandemic strain of H1N1 (pH1N1) virus are available.

**Materials and Methods:**

Surveillance of influenza-related medical encounter data of active duty military service members stationed in the United States during the period of April–October 2009 with comparison of pH1N1-confirmed cases and location and date-matched controls. Crude odds ratios (OR) and VE estimates for immunized versus non-immunized were calculated as well as adjusted OR (AOR) controlling for sex, age group, and history of prior influenza vaccination. Separate stratified VE analyses by vaccine type (trivalent inactivated [TIV] or live attenuated [LAIV]), age groups and hospitalization status were also performed. For the period of April 20 to October 15, 2009, a total of 1,205 cases of pH1N1-confirmed cases were reported, 966 (80%) among males and over one-half (58%) under 25 years of age. Overall VE for service members was found to be 45% (95% CI, 33 to 55%). Immunization with prior season's TIV (VE = 44%, 95% CI, 32 to 54%) as well as LAIV (VE = 24%, 95% CI, 6 to 38%) were both found to be associated with protection. Of significance, VE against a severe disease outcome was higher (VE = 62%, 95% CI, 14 to 84%) than against milder outcomes (VE = 42%, 95% CI, 29 to 53%).

**Conclusion:**

A moderate association with protection against clinically apparent, laboratory-confirmed Pandemic (H1N1) 2009-associated illness was found for immunization with either TIV or LAIV 2008–09 seasonal influenza vaccines. This association with protection was found to be especially apparent for severe disease as compared to milder outcome, as well as in the youngest and older populations. Prior vaccination with seasonal influenza vaccines in 2004–08 was also independently associated with protection.

## Introduction

Influenza is a common infection among military personnel who are frequently exposed to a variety of respiratory pathogens in crowded living conditions, stressful working environments and during deployments [1]. An annual influenza vaccination policy was implemented for active duty personnel during World War II, which subsequently led to the prevention of large influenza epidemics in military personnel [2]. However, influenza outbreaks of novel strains have occurred, such as the previous appearance of a “swine influenza” A/H1N1 strain among soldiers at Fort Dix, New Jersey, in early 1976, [3] as well as the ongoing pandemic caused by a novel influenza A/H1N1 (pH1N1) virus [4]. World governments and the scientific community have renewed concerns about a lack of population immunity as well as the reported lack of cross-protective immunity from seasonal influenza vaccines [5].

Trivalent inactivated vaccine (TIV) formulations have been in use by the US military for the past six decades [2]. Live attenuated influenza vaccine (LAIV) was added during the 2003–04 influenza season. Since the introduction of LAIV, Department of Defense (DoD) policy has called for preferential use of LAIV over TIV stemming from vaccine shortages during the 2003–2004 influenza season and reported benefits in the young, healthy recruit populations [6], [7]. Recent clinical trials, [8], [9] as well as DoD-based analyses of influenza, influenza-like illnesses and pneumonia-related healthcare encounters, [6], [7] suggest that TIV is more efficacious against laboratory-confirmed influenza among civilians as well as among highly-immunized military service members. Conversely, previously published AFHSC data also suggest that LAIV may be just as effective as TIV among vaccine-naïve personnel [7]. The primary objective of this effort was to provide an interim assessment of the effectiveness of a single season's (2008–2009) influenza vaccine against clinically-apparent, laboratory-confirmed pH1N1-associated illness. The results of this study will help to develop a mechanism for systematically tracking and assessing vaccine effectiveness (VE) for the newly available monovalent H1N1 pandemic vaccine and seasonal influenza vaccines of the future.

## Materials and Methods

*Ethics Statement: The AFHSC has been directed by military authorities to conduct public health surveillance of respiratory infectious diseases and evaluation of related protection measures. According to 45 CFR 46.101/102, this activity does not constitute research, thus, institutional review board examination was not required. No external (non-DoD) funding was used to conduct this investigation, and contents have been cleared for public release by the US Army Public Health Command (Provisional).*

The surveillance population of interest was all active component service members (as opposed to those in the National Guard or Reserves) stationed in the United States at some point during the period of April 20 through October 15, 2009. Data were obtained from the Defense Medical Surveillance System (DMSS), a large relational database that contains longitudinal data including demographic characteristics, occupations, immunizations and medical encounters for US military service members [10]. Data collection begins at the time of entry into service and continues through the military career. Certain medical conditions of military relevance, including laboratory-confirmed influenza, are submitted through electronic notifiable disease reporting systems using case definitions established by the Armed Forces Health Surveillance Center (AFHSC) and are part of the DMSS data [11].

Reporting criteria for influenza was defined as a clinically-apparent illness (fever, cough and/or sore throat) which was confirmed by polymerase-chain reaction (PCR). Reports of confirmed influenza from Army, Air Force and Navy (including Marine Corps and US Coast Guard) reporting systems were included as part of the DMSS data.

Cases were defined as active component service members with a laboratory-confirmed pH1N1-associated illness reported through one of the service-specific notifiable disease reporting systems. Controls were defined as active component service members who reported to the same military treatment facility as their date-matched case with a diagnosis of a musculoskeletal (*International Classification of Diseases*, *Ninth Revision*, *Clinical Modification* (ICD-9-CM) = 700–739, 810–848, or V54) or a mental health encounter (ICD-9-CM = 700–739, 810–848, or V54) and no documented respiratory problems (ICD-9-CM = 001–139, 320–326, 380–382, 460–519, 780.6, 780.7, 786, or 787.0) during the medical visit. The control's medical encounter had to occur within 3 days of the case's medical encounter. A maximum of four controls were matched to each case.

Immunization data from DMSS were used to determine whether cases and controls received any influenza vaccination during the influenza season of August 1, 2008 through July 31, 2009. Subjects who received an influenza vaccine at least 14 days prior to the date of their qualifying medical encounter were considered immunized; all others (those immunized less than 14 days prior to medical encounter, those vaccinated after the medical encounter, or those not vaccinated with the current seasonal influenza vaccine) were considered non-immunized for the purposes of this evaluation.

Crude odds ratios (OR) were calculated for comparison of cases to controls by multiple factors including sex, age group (<25, 25 to 29, 30 to 39, 40 and over), race-ethnicity (White, Black, Hispanic, Asian/Pacific Islander, American/Alaskan Indian, Other/unknown), service (Army, Air Force, Coast Guard, Navy, Marine Corps), history of underlying medical conditions (required at least one prior medical encounter with a primary diagnosis of asthma, chronic obstructive pulmonary disease, diabetes, chronic renal disease, cancer, circulatory system conditions, or nervous system conditions), pregnancy, non-influenza vaccines administered 0–30 days prior to the influenza vaccine, and history of prior influenza vaccination (yes/no). Adjusted OR (AOR) for vaccination status was calculated using conditional logistic regression adjusting for sex, age group, and history of prior influenza vaccination. Separate stratified VE analyses by age group, vaccine type (TIV and LAIV) and hospitalization status were performed and VE estimates were adjusted for sex, age group (except for the age stratified analysis), and history of prior vaccination. VE was defined as (1 – OR *100) as previously published [12] the adjusted odds ratios for LAIV and TIV in the vaccine stratified analysis were tested for homogeneity using a conditional logistic regression model. All analyses were performed using SAS 9.1.3 (SAS Institute, Cary, North Carolina, USA).

## Results

During the period April 20, 2009 to October 15, 2009, a total of 1,205 clinically-apparent, laboratory-confirmed pH1N1-associated illnesses were reported. Case subjects were similar to controls with the exception of age; mean and median age for cases and controls was found to be 25.3 and 23 years compared to 30.2 and 28 years, respectively, Cases were also noted to have received fewer vaccines in prior years than controls (Table 1). Controls were more likely to have a history of an underlying medical condition compared to cases (46% versus 24%, respectively). Regardless of case-control status, vaccinated subjects were also more likely to have a history of an underlying medical condition compared to unvaccinated subjects (For Cases: 27% versus 11%; For Controls: 47% versus 36%). However, since having history of an underlying medical condition was highly correlated with age and receipt of prior influenza vaccine during 2004–08 (two variables already included in the adjusted model), this variable had no effect on the VE estimate when added to the model and was therefore not include in the final adjusted model. Additionally, no differences were seen in the percent of cases and controls who received non-influenza vaccines within the 30-days prior to the influenza vaccine and none of the subjects were pregnant. Cases were distributed over a wide geographic range with most (74%) reported in six states (Texas, n = 511; California, n = 128; South Carolina, n = 73; Florida, n = 68; North Carolina, n = 58; and, Missouri, n = 49) and the remaining 318 cases distributed among 26 additional states (AK, AR, AZ, CO, DC, GA, HI, IL, KS, KY, LA, MA, MD, ME, MS, ND, NE, NJ, NM, NY, OH, OK, SD, VA, WA and WY).

**Table 1:** Univariate Analysis and Characteristics of pH1N1 Cases and Controls.

	Cases	Controls	Crude OR (95% CI)
**Sex**			
Male	966 (80.2)	3584 (74.5)	1.39 (1.19–1.63)
Female	239 (19.8)	1226 (25.5)	Ref
**Age Group**			
<25	696 (57.8)	1572 (32.7)	Ref
25–29	289 (24.0)	1058 (22.0)	0.59 (0.50–0.70)
30–39	151 (12.5)	1398 (29.1)	0.23 (0.19–0.28)
40+	69 (5.7)	782 (16.3)	0.18 (0.14–0.24)
**Race-ethnicity**			
White	691 (57.3)	3054 (63.5)	Ref
Hispanic	151 (12.5)	496 (10.3)	1.36 (1.11–1.66)
Black	235 (19.5)	884 (18.4)	1.19 (1.00–1.41)
Asian/Pacific Islander	74 (6.1)	172 (3.6)	1.92 (1.44–2.56)
American Indian/Alaskan Native	14 (1.2)	53 (1.1)	1.19 (0.66–2.15)
Other/Unknown	40 (3.3)	151 (3.1)	1.18 (0.82–1.68)
**Service**			
Army	445 (36.9)	1908 (39.7)	Ref
Air Force	527 (43.7)	2012 (41.8)	1.56 (1.18–2.07)
Navy	88 (7.3)	295 (6.1)	1.81 (1.22–2.70)
Marine Corps	130 (10.8)	541 (11.2)	1.25 (0.81–1.92)
Coast Guard	15 (1.2)	54 (1.1)	1.55 (0.65–3.71)
**Number of prior vaccinations**			
0	443 (36.8)	872 (18.1)	Ref
1+	762 (63.2)	3938 (81.9)	0.33 (0.28–0.38)

Note: OR = Odds Ratio.

Overall, a moderate association with protection with any 2008–09 seasonal influenza vaccine was observed with a VE of 45% (95% CI, 33 to 55%) (Table 2). Age-stratified analyses revealed an independent, age-associated effect. Younger and older individuals (<25 years, VE = 50%; 40+ years, VE = 55%) exhibited a markedly higher VE estimate than those 25–29 years (VE = −6%) or 30–39 years (VE = 9%) (Table 3). In addition, prior vaccination in 2004–08 timeframe (VE = 41%, 95% CI, 29 to 51%) was also significantly associated with protection (Table 4).

**Table 2:** Crude and Adjusted OR for Any Vaccine Received in 2008–2009.

	Cases, n (%)	Controls, n (%)	Crude OR (95% CI)	Adjusted OR (95% CI)*	Vaccine Effectiveness (95% CI)
**Influenza Vaccine**					
Yes	956 (79.3)	4291 (89.2)	0.43 (0.36–0.51)	0.55 (0.45–0.67)	45% (33 to 55%)
No	249 (20.7)	519 (10.8)	Ref	Ref	Ref

*Adjusted for sex, age group, and number of prior vaccinations.

**Table 3:** Crude and Adjusted OR for Specific Age-Groups for Any Vaccine Received in 2008–2009.

	Cases, n (%)	Controls, n (%)	Crude OR (95% CI)	Adjusted OR (95% CI)**	Vaccine Effectiveness (95% CI)
**Age group<25 years**					
Influenza Vaccine					
Yes	507 (72.8)	1344 (85.5)	0.45 (0.37–0.57)	0.50 (0.40–0.63)	50% (37 to 60%)
No	189 (27.2)	228 (14.5)	Ref	Ref	Ref
**Age group 25–29 years**					
Influenza Vaccine					
Yes	259 (89.6)	959 (90.6)	0.89 (0.58–1.37)	1.06 (0.68–1.67)	−6% (−67 to 32%)
No	30 (10.4)	99 (9.4)	Ref	Ref	Ref
**Age group 30–39 years**					
Influenza Vaccine					
Yes	136 (90.1)	1272 (91.0)	0.90 (0.51–1.58)	0.91 (0.51–1.63)	9% (−63 to 49%)
No	15 (9.9)	126 (9.0)	Ref	Ref	Ref
**Age group 40+**					
Influenza Vaccine					
Yes	54 (78.3)	716 (91.6)	0.33 (0.18–0.62)	0.45 (0.22–0.93)	55% (7 to 78%)
No	15 (21.7)	66 (8.4)	Ref	Ref	Ref

**Adjusted for sex and number of prior vaccinations.

**Table 4:** Crude and Adjusted OR for Service Members with a Documented History of Receiving Previous Influenza Vaccines.

	Cases, n (%)	Controls, n (%)	Crude OR (95% CI)	Adjusted OR (95% CI)***	Vaccine Effectiveness (95% CI)
**Number of prior vaccinations (2004–2008)**					
0	443 (36.8)	872 (18.1)	Ref	Ref	Ref
1+	762 (63.2)	3938 (81.9)	0.33 (0.28–0.38)	0.59 (0.49–0.71)	41% (29 to 51%)

Note: OR = Odds Ratio.

***Adjusted for sex and age group.

In the stratified analysis of vaccine-specific effectiveness, both TIV and LAIV were found to be associated with protection (Table 5). The adjusted VE estimate ranged from 44% (95% CI, 32 to 54%) to 24% (95% CI, 6 to 38%) for TIV and LAIV, respectively. The association with protection provided by TIV was not found to be statistically significantly different from LAIV (p-value = 0.3206).

**Table 5:** Vaccine-specific Crude and Adjusted OR for Cases Received the 2008–2009 Trivalent Influenza Vaccine (TIV) or Live Attenuated Influenza Vaccine (LAIV).

	Cases, n (%)	Controls, n (%)	Crude OR (95% CI)	Adjusted OR (95% CI)*	Vaccine Effectiveness (95% CI)
**Vaccine Type**					
**TIV**					
Yes	440 (63.9)	2063 (79.9)	0.44 (0.37–0.53)	0.56 (0.46–0.68)	44% (32 to 54%)
No	249 (36.1)	519 (20.1)	Ref	Ref	Ref
**LAIV**					
Yes	505 (67.0)	2166 (80.7)	0.49 (0.41–0.58)	0.76 (0.62–0.94)	24% (6 to 38%)
No	249 (33.0)	519 (19.3)	Ref	Ref	Ref

Note: OR = Odds Ratio.

*Adjusted for sex, age group, and number of prior vaccinations.

**Figure 1:**
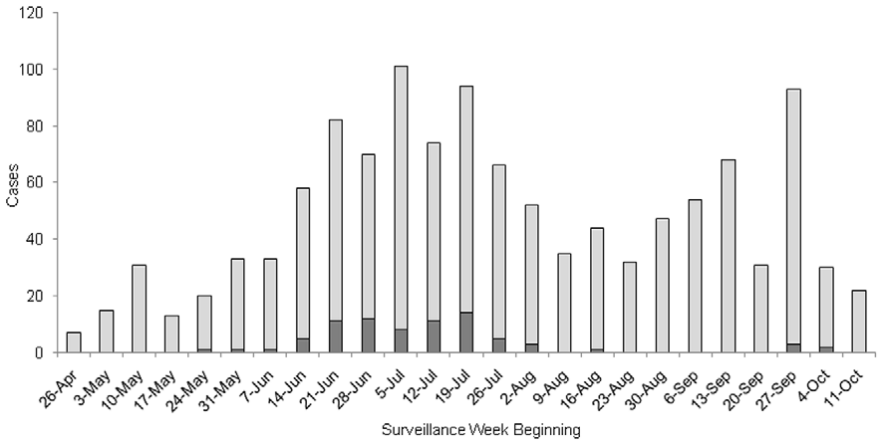
Number of Hospitalized and Non-hospitalized pH1N1 Cases by Week. 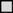
 Light Bars = Non-Hospitalized Cases. 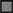 Dark Bars = Hospitalized Cases.

A total of 78 (6.5%) of the 1,205 cases were hospitalized (Figure 1). Over 88% of the hospitalizations occurred over a seven-week period in June and July of 2009. Assessment of VE with regards to disease severity showed there to be a higher association with protection against more severe outcomes (e.g., for those hospitalized). This association with protection was greater among hospitalized cases (62%) compared to non-hospitalized cases (42%) (Table 6).

**Table 6:** Crude and Adjusted OR for Hospitalized and Non-hospitalized Cases and Controls.

	Cases, n	Controls, n	Crude OR (95% CI)	Adjusted OR (95% CI)*	Vaccine Effectiveness (95% CI)
**Hospitalized, any Vaccine**					
Yes	44	276	0.15 (0.08–0.29)	0.38 (0.16–0.86)	62% (14 to 84%)
No	34	36	Ref	Ref	Ref
**Non-hospitalized, any Vaccine**					
Yes	912	4015	0.48 (0.39–0.57)	0.58 (0.47–0.71)	42% (29 to 53%)
No	215	483	Ref	Ref	Ref

Note: OR = Odds Ratio.

*Adjusted for sex, age group, and number of prior vaccinations.

## Discussion

The results of this assessment suggest there is an association with protection from the northern hemisphere 2008–09 seasonal influenza vaccine against clinically-apparent, laboratory-confirmed pH1N1-associated illness among active component US military service members. This association with protection may be more apparent for hospitalized (more severe) outcomes and warrants further investigation. Our findings further complement recent reports among civilian populations in Mexico [13], [14] and among health care professional in Ontario, Canada [15] that demonstrate moderate to high (35 to 73%) vaccine effectiveness against pH1N1-associated illness from seasonal influenza vaccination. A very recent report from a Singaporean prospective study [16] indicated a marked increased risk of pH1N1 infection in military personnel and has also provided further evidence of an association with protection from seasonal influenza vaccines among military personnel when compared to civilian populations.

In contrast, published findings from Australia, [17] and three US-based studies [18]–[20] showed either negligible association with protection (overall VE = −10 to 3%, 95% CI, −56 to 40%). Moreover, one recently published study out of Canada documented an increased risk for medically-attended, laboratory-confirmed pH1N1 after receipt of seasonal vaccine (VE = −68%, 95% CI, −174 to 3%) [21]. The health care encounter-related findings of our vaccine effectiveness assessment also expand further on recently published laboratory-based serologic studies of the effect of TIV vaccines against pH1N1 [22]–[24].

Our data also suggests that prior receipt of TIV or LAIV induces an association of protection against pH1N1-associated illness. This may reflect “priming” of the humoral immune system with influenza vaccine as demonstrated in immunologically-naïve children [25], [26]. Similar findings have also been observed in US military populations where the influenza vaccine increased the effectiveness of preventing pneumonia and influenza morbidity among vaccine-naïve service members compared to service members routinely immunized [7]. Our findings also expand on the observations by Ohmit, et al, [9] and Monto, et al, [8] in their prospective, randomized, double-blind, placebo-controlled, 4-year study of efficacy demonstrating that TIV offers a higher degree of protection against laboratory-confirmed influenza in years 1 (2004–05) and 4 (2007–08) of their study.

Like the US CDC's recently published US serologic data [22]–[24] our findings also strongly suggest an age-related association with protection. However, it appears that any association with protection may actually occur in those as young as 17 to 24 years of age. An unexpected finding of our study was the increased association with protection in those 40 years of age and older, perhaps reflecting an association with previous vaccine exposure and/or natural infection with other human H1N1 viruses in the setting of crowded living conditions prevalent in the military environment or in the population old enough to have been exposed to 1918-like H1N1 viruses [1], [27], [28], [29]. There are reports of cross-reactive protection induced by vaccination and infection with virus strains that are divergent between, and within, influenza A virus subtypes in animal models. It seems likely that, in addition to induced hemagglutinin (HA) strain-specific antibody responses, that cross-reactive epitopes on the HA and neuraminidase (NA) external proteins, as well as, immune responses to epitopes on internal proteins can contribute to protection against influenza [30]–[35]. In addition to these specific epitopes, other studies have suggested that neutralizing capability depends also on the affinity and avidity of the antibodies such that quality may be more of a factor than quantity alone [36]–[39]. To what degree host-specific, genetically-determined immune responses further confound vaccine effectiveness (or efficacy) has not been adequately studied and may represent an important biological/host confounder which is difficult to address in epidemiologic studies such as this.

Additional findings from our study support the notion that vaccination with seasonal influenza vaccines in the preceding four years (2004–08) also conferred a certain degree of protective immunological memory relevant to the new viral strain. Indeed, it has been shown in previous studies that both humoral and cell-mediated immune (CMI) responses may contribute to protection in influenza-vaccinated persons. In animal studies, the role of CMI in viral clearance and host survival has been shown and increasing evidence is available regarding T cell-mediated immune responses in humans after natural infection or vaccination [40], [41]. Thus, it is reasonable to think that CMI plays a significant role and that cross-protective CMI to pH1N1 virus may actually exist in individuals who have been frequently immunized and/or exposed to seasonal influenza [42]. As recently described by Greenbaum, et al, [43] it is also possible that some degree of pre-existing “memory” conferred by exposure to T-cell epitopes, similar to those found in previously circulating H1N1 strains in the past 20 years (1988–2008), may indeed work to elicit increased immunity of adults. This observation may explain the cumulative enhanced benefit of multiple prior influenza vaccines overlapping with increased potential seasonal exposures in older subjects.

There are several limitations with this study. First, tobacco exposure (e.g., smoking), an important co-factor in increasing the risk for influenza infection/disease, was not addressed in this study. There is animal, laboratory-based [44] and human epidemiologic-based evidence [45] which strongly suggests smoking as an important factor in predisposing to influenza infection and/or pneumonia. It is possible that lower rates of tobacco use among older military populations contrasted with higher use among younger military personnel outside of basic training (where tobacco use is more controlled) may have been an important confounder not adjusted for in this study.

Second, the vaccination status was based upon electronic data and relied on reporting by the vaccinating health care providers. The possibility of misclassification of vaccine status exists. However, we believe this to be minimal and non-differential between the cases and controls. Furthermore, this potential limitation would bias our results towards the null, therefore underestimating the overall effectiveness of the vaccines.

Third, it is possible that misclassification of cases into the control group may have occurred. Since laboratory testing requirements for pH1N1 confirmation have changed over the time period of this study (starting with universal testing to only testing severe cases), and since physicians may not have requested confirmatory testing for all suspected cases, the possibility exists that a control subject may have been infected with pH1N1 but did not get recorded as a laboratory-confirmed case. In order to decrease this risk of misclassification we excluded from our potential control pool anyone who had a wide range of respiratory-associated symptoms or diagnoses during their qualifying medical encounter. However, if this misclassification did occur we expect it would be non-differential in nature and, again, bias our results toward the null.

Fourth, both cases and controls were highly vaccinated; 80% and 89%, respectively. Thus, it is possible that the minority of the service members who did not receive the 2008–09 seasonal vaccine may have differed in the risk for influenza if they suffered from predisposing, co-morbid conditions which may have increased their risk of infection and/or illness as previously described [46]. However, similar to findings from observational studies among the elderly, we actually found that vaccinated subjects were more likely to have history of an underlying medical condition compared to unvaccinated subjects [47]–[49]. This could have potentially biased our study results towards the null (e.g. less VE), however, when added to the model we found this had no effect on the VE estimates due to the high correlation with age and prior receipt of an influenza vaccine.

Lastly, there are a number of studies that clearly illustrate the inherent bias in assessing influenza vaccine effectiveness when conducting observational, cross-sectional studies such as ours [50], [51]. Inherent biases in case ascertainment and access to care may have taken place, however, we feel these potential biases were minimized due to our study population. The active component military population receives universal health care coverage at military treatment facilities regardless of the nature of their underlying conditions or presenting medical symptoms (e.g., equal access to care for respiratory and non-respiratory complaints) and thus, would not have influenced our results in a significant manner.

Our finding of a greater association with protection against severe illness (e.g., hospitalization) suggests that the northern hemisphere 2008–09 influenza vaccine may have a more significant impact against overt pH1N1-associated illness compared to subclinical infection. This warrants further analysis with a larger sample size looking at age, sex, race and other factors, specifically for hospitalized individuals. Prospective monitoring of health care outcomes which may be indicative of severe pH1N1-associated illness, such as severe acute respiratory infections (SARI) and pneumonia is being implemented among all beneficiaries of the military health system.

Ongoing, systematic evaluations of seasonal and pH1N1-specific vaccination programs are critical to assess the overall public health impact of these interventions. Our data supports the importance of continued immunization coverage for all populations as recently recommended by the CDC's Advisory Committee on Immunization Practices [52]. Expanded assessment of vaccine effectiveness among high-risk recruit populations who are immunologically-naïve and who traditionally sustain higher rates of acute respiratory infections, [27] as well as, among young children and high-risk adults, are indicated and may further refine understanding of biological diversity based on age, sex and background disease states. In addition, the role of multiple previous influenza vaccines on immune response and vaccine efficacy/effectiveness deserves further investigation.

Further studies of the potential association between prior seasonal and pH1N1-specific influenza vaccinations (either single- or multi-year reception) and seasonal as well as pH1N1-associated illnesses are needed and should include prospective cohort as well as retrospective case-control studies using sentinel surveillance data [17], [53], [54]. In addition, future immunologic assessments of any age-related protective effect (possibly due to the presence of natural infection or vaccine-induced cross-reactive antibodies) should also be conducted to further elucidate this relationship. A greater number of cases with a broader, older age representation are needed to study these hypotheses more thoroughly. In addition, the role of sex differences on vaccine immune responses and associated efficacy/effectiveness estimations needs to be evaluated through epidemiologic studies [55].

In summary, a moderate association with protection against clinically-apparent, laboratory-confirmed pH1N1-associated illness was found for immunization with either TIV or LAIV seasonal influenza vaccines. This association with protection was greater for severe disease as compared to milder outcomes. There was also a greater association with protection in the youngest (<25 years) and oldest (40+ years) compared to those 25 to 39 years. Prior vaccination in the 2004–08 timeframe was also independently associated with protection. Cross-protective immunity, as a result of natural influenza infections or prior influenza immunization in the military setting, may play a role in conferring a certain degree of enhanced host immunity as exposure takes place with each subsequent influenza season strain(s). Therefore, it is important to examine host-specific, genetically-determined factors in future assessments of influenza vaccine efficacy and/or effectiveness.
